# Does Chronic Unpredictable Stress during Adolescence Affect Spatial Cognition in Adulthood?

**DOI:** 10.1371/journal.pone.0141908

**Published:** 2015-11-18

**Authors:** Lauren E. Chaby, Michael J. Sheriff, Amy M. Hirrlinger, James Lim, Thomas B. Fetherston, Victoria A. Braithwaite

**Affiliations:** 1 Center for Brain, Behavior, and Cognition, Pennsylvania State University, University Park, Pennsylvania, United States of America; 2 Department of Ecosystem Science & Management, Pennsylvania State University, University Park, Pennsylvania, United States of America; 3 Huck Institutes of the Life Sciences, Pennsylvania State University, University Park, Pennsylvania, United States of America; 4 Department of Biology, Pennsylvania State University, University Park, Pennsylvania, United States of America; 5 College of Agriculture, Pennsylvania State University, University Park, Pennsylvania, United States of America; Technion - Israel Institute of Technology, ISRAEL

## Abstract

Spatial abilities allow animals to retain and cognitively manipulate information about their spatial environment and are dependent upon neural structures that mature during adolescence. Exposure to stress in adolescence is thought to disrupt neural maturation, possibly compromising cognitive processes later in life. We examined whether exposure to chronic unpredictable stress in adolescence affects spatial ability in late adulthood. We evaluated spatial learning, reference and working memory, as well as long-term retention of visuospatial cues using a radial arm water maze. We found that stress in adolescence decreased the rate of improvement in spatial learning in adulthood. However, we found no overall performance impairments in adult reference memory, working memory, or retention caused by adolescent-stress. Together, these findings suggest that adolescent-stress may alter the strategy used to solve spatial challenges, resulting in performance that is more consistent but is not refined by incorporating available spatial information. Interestingly, we also found that adolescent-stressed rats showed a shorter latency to begin the water maze task when re-exposed to the maze after an overnight delay compared with control rats. This suggests that adolescent exposure to reoccurring stressors may prepare animals for subsequent reoccurring challenges. Overall, our results show that stress in adolescence does not affect all cognitive processes, but may affect cognition in a context-dependent manner.

**Highlights**

- Rats were reared with or without chronic unpredictable stress in adolescence.
- In adulthood, spatial cognitive abilities were tested in a radial arm water maze.
- Prior-stressed rats began searching faster in the maze after an overnight delay.
- Prior stress may facilitate faster action in challenging situations.
- Prior stress did not affect learning, reference or working memory, or retention.

## Introduction

Spatial cognition can increase foraging efficiency, enhance the ability to locate mates, improve parental care, help animals minimize their exposure to danger, and, in humans, predict life outcomes [1], [2], [3], [4]. Spatial ability is the set of cognitive processes that allow an individual to recall and manipulate information about spatial objects in their environment, and includes many distinct cognitive processes including learning, memory, and problem solving using spatial information [5], [1]. In humans, spatial ability can predict educational-vocational track [6]; adolescents with poor spatial ability show reduced learning [7] and are less likely to obtain a career in science, engineering, technology, or math [4]. The cues or types of spatial abilities animals rely on can be shaped by their environment [8], [9] and harsh environments or seasonal changes can modulate spatial abilities and their underlying neural physiology [10], [11]. Over time, exposure to a stressful environment can impair spatial learning and memory [12], [13] and affect place-object memory and object recognition [14]. Stress exposure can alter spatial ability both at the time of exposure and long after the stressful stimulus has been removed, however, the characteristics of these effects can vary across an individual’s lifespan [15], [16]. Understanding the nature of these processes could inform us about the functionality of such changes within an ecological context, as well as shedding light on life outcomes in human populations.

The effects of exposure to stress on spatial ability appear to be dependent upon age at exposure [17], [18]; spatial abilities can be impaired by exposure to stress during prenatal development [19], [20] or in the first few weeks of postnatal life [16]. It has been suggested that exposure to stress in early life may decrease reliance on spatial learning and enhance emotional learning to prepare developing individuals for an uncertain or high-stress environment later in life [16]. Adolescence may also be a period of vulnerability to stress-induced changes in spatial abilities *sensu* [21], [22]. McCormick et al. [23] showed that adult Long-Evans rats exposed to chronic social stress during adolescence spend less time investigating a familiar object if it is moved to a new location compared with unstressed rats, indicating reduced hippocampal-dependent object memory. Similarly, compared with unstressed conspecifics, mice exposed to social instability stress in adolescence spend less time exploring a novel arm of a familiar maze when tested 12 months after chronic stress exposure has ceased, suggesting possible decreases in hippocampal-dependent spatial memory [24]. Sterlemann et al. [24] also showed that chronic adolescent-stress can cause an impairment in spatial learning that is apparent only after a delay; mice exposed to social instability stress in adolescence took longer to find an escape platform in a Morris water maze in the last 5 of 12 trials, but showed no difference in earlier trials or in a hippocampus-independent learning task.

Despite the clear ramifications of experiencing stress during adolescence [25], its long-term effects on spatial ability are underexplored and the specific cognitive processes that are affected remain unclear. Differences in spatial ability could be driven by changes in spatial-reference memory, spatial-working memory, or long-term retention of spatial cues. Investigating which processes are affected could inform how deficits in spatial ability may affect other behavioral domains (e.g. foraging, mate location, etc.) and which aspects of cognition may be most vulnerable to lasting changes caused by exposure to stress. For example, deficits caused by adolescent-stress in learning and object memory have only been detected after a time delay [23], [24], [25] and so may be commonly mediated by impaired retention (reference memory).

Here we tested the hypothesis that rats exposed to chronic adolescent-stress would exhibit deficits in spatial abilities that would be mediated by a subset of cognitive processes, including differences in retention. Specifically, we evaluated several aspects of spatial ability: spatial learning, reference and working memory, and long-term retention of visuospatial cues in a radial arm water maze (RAWM; [26], [27]), in rats that experienced chronic stress during adolescence and rats reared in unstressed conditions.

## Methods

### Animals and housing

**Fig 1:**
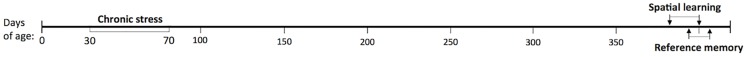
Timeline of manipulations.

Male Sprague-Dawley rats (n = 24) were obtained at 21 days of age from Harlan Laboratory in Frederick, Maryland. Following transport, rats were given 7 days to acclimate before handling and experimental procedures began (see Fig 1 for timeline). Animals were randomly assigned to pair-housing in plastic cages (20cm x 26cm x 45cm) with wood chip bedding, two pine wood chews, and two 7.6cm diameter PVC tubes. Cages were changed weekly; wood chews and PVC tubes were replaced when visibly soiled. Standard rat chow (LabDiet^®^ 5001, 23% protein) and tap water were available *ad libitum* unless otherwise noted. Rats were kept at 20–21°C and 40–45% relative humidity on a 12:12 reversed light/dark cycle; the dark phase was 0900h-2100h. To control for circadian rhythms, all testing began a minimum of 2 hours after the beginning of the dark phase and was completed within 8 hours. Testing order was pseudo-randomized; the order in which individual rats were tested varied each day with treatment groups balanced across the first and last hours of the testing session. Body mass was monitored weekly as an indicator of health throughout adolescence and testing in adulthood. Experiments were approved by the Pennsylvania State University Institutional Animal Care and Use Committee (IACUC), protocol #44459.

### Chronic unpredictable stress

Pair-housed rats were randomly assigned to either the adolescent-stress treatment (n = 12) or to the unstressed control group (n = 12). Rats in the adolescent-stress treatment were exposed to physical, social, and predation stressors from 30–70 days of age [28], [29]. Though some suggest that adolescence concludes at approximately 55 days of age in male rodents, many studies have included a postpubertal “sub-adult” period to cover the entire ontogenetic window of adolescence, 28–80 days of age [23], [30], [31]. Adolescent exposure to the chronic unpredictable stress paradigm used here has previously been shown to induce behavioral and cognitive changes in adulthood that correspond to the current age at testing [28], [29].

- Physical stressors: (1) Housed in a cage 25% smaller than the home cage for 4 hours, (2) housed with damp bedding for 6 hours, (3) home cage tilted 30° for 6 hours.
- Social stressors: (1) Housed individually for 1 hour, (2) crowding with 2 rat pairs in a standard cage for 4 hours, (3) exposed to bedding from older conspecifics for 12 hours.
- Predation stressors: Exposed for 30 minutes to (1) a continuously moving taxidermied bobcat [32], (2) *Felis catus* fur, (3) large cat vocalizations.

Each of the three types of stress were represented twice per week, leaving one rest day. Stressors were presented unpredictably during both phases of the light/dark cycle, but balanced such that within each week rats encountered approximately three stressors between 0–1200h and three stressors between 1200h-2400h. To account for the additional handling and cage changes required to enact the stressors, rats in the control group experienced biweekly handling sessions and cage changes that coincided with stressors requiring new cages. During stress treatments rats were continuously pair-housed unless specified.

### Radial arm water maze

**Fig 2:**
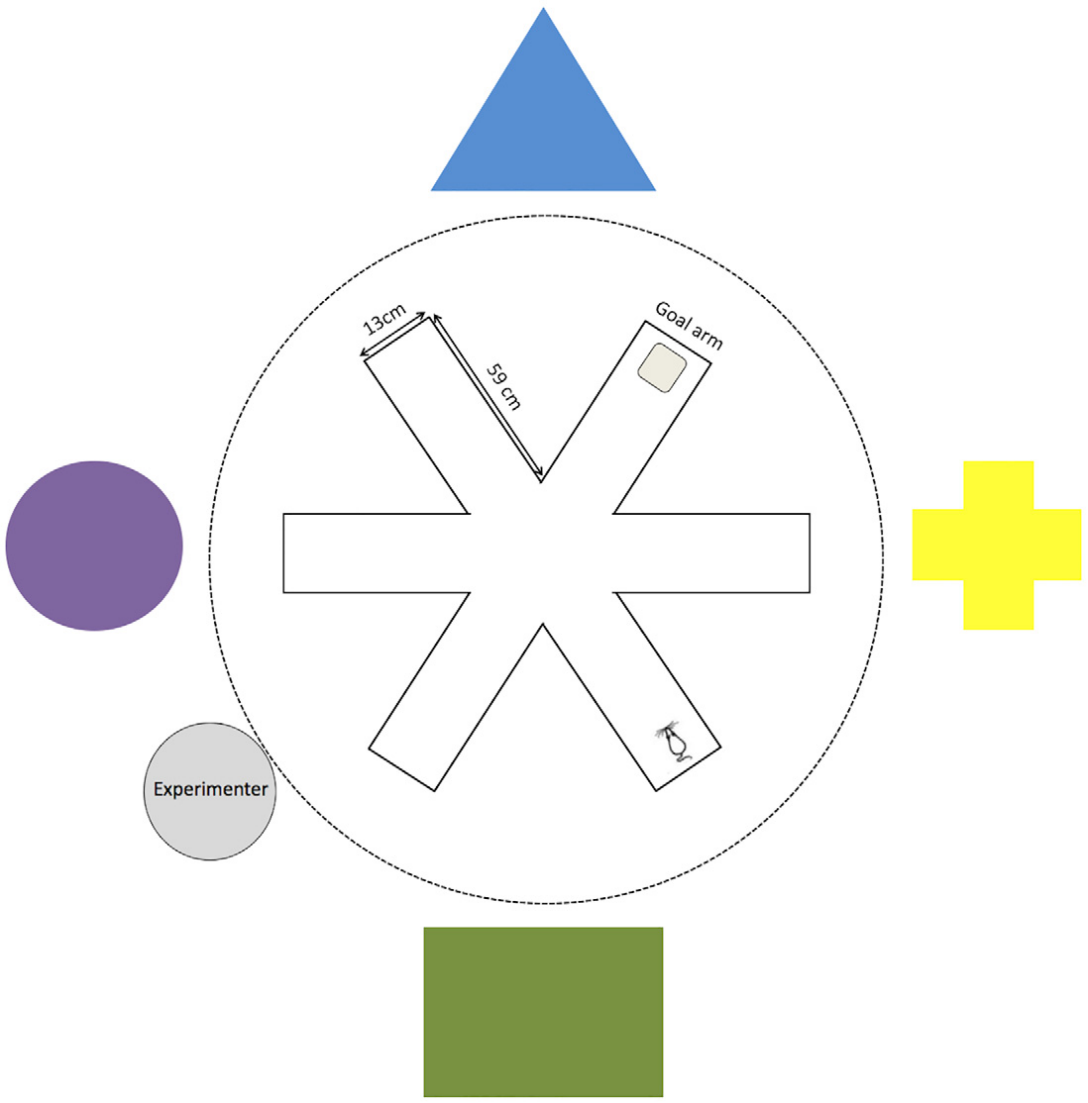
Radial arm water maze and visual cue schematic (not to scale). Goal arm was counterbalanced across treatment condition. For each rat, the goal arm remained the same throughout all trials, but the starting arm was randomized so that rats had to learn a spatial location, and could not rely on a motor rule.

Starting 10.5 months after completion of the stress paradigm, rats were tested in a six-arm radial water maze surrounded by an opaque white plastic curtain (Fig 2). Visual cues were attached to the curtain in each cardinal compass direction. The experimenter remained in the same position throughout testing to maintain consistency in visual cues [33]. The water was constantly monitored and maintained between 24–25°C at a depth of 43cm [27]. The water was made opaque using non-toxic Tempera paint [34].

After every exposure to the water maze, rats were briefly towel-dried and transferred to a holding cage that contained a heating pad under dry towels. A heating lamp warmed one side of the holding cage, allowing the rat an option to escape the heat. Rats remained in the holding cage until dry and behaving normally (at least 10–15 minutes). Rats were observed daily following exposure to the water maze; no signs of dehydration, abnormal vocalizations, decreased weight or appetite, postural abnormalities, or labored respiration were detected. Between each trial, any fecal matter was removed from the water with a dip net. During each day of trials, partial water changes were used to maintain cleanliness of the maze. At the end of each day of trials, the maze was drained, wiped clean, and dried thoroughly.

#### Spatial abilities

We tested each rat’s ability to associate a maze arm with a platform that allowed them to exit the water (procedures are modeled on [27]). Each rat was tested on two sequential days, with 15 trials per day, between 387 to 400 days of age. To avoid fatigue from consecutive trials, the training schedule was spaced by grouping rats into two waves that were tested on alternating days (balanced by treatment), by allowing rats to fully recover between trials, and by limiting an individual rat’s testing time to 3 hours per day [27]. The arm containing the platform (the goal arm) remained the same for each rat and was counterbalanced across stress condition. For the initial phase of the water maze, the trials alternated such that the platform was either “invisible” just below the surface of the opaque water or “visible” 2cm above the surface; the first trial of each day was always “visible” in order to facilitate learning of the platform location [27]. After the second training day, the platform was “invisible” in all subsequent exposures to the maze. To begin each trial, a rat was placed at the end of an arm that did not contain the platform. The starting arm was randomized so that rats had to learn the spatial location of the platform and could not rely on a motor rule, e.g. first turn to the left. During the first exposure to the maze, if the rat had not located the platform 1 minute after entering the water, or after 2 minutes on all subsequent learning trials, a hand was placed behind the rat to guide it through the water in the direction of the platform. After the rat was guided to or located the platform, it was removed from the maze after all four feet of the rat were on the platform for 15 seconds. For the reference and working memory trials, if a rat had not located the platform within 2 minutes it would be removed, however, no rat failed to locate the platform within 2 minutes.

To assess spatial ability, we measured the latency to find the platform (contact the platform with a paw or nose), search time (latency to find the platform–latency to first arm entry), and the number of arm entries. An arm entry was defined as having all four paws within a maze arm. We quantified reference memory errors (entering an arm other than the goal arm) and working memory errors (any subsequent re-entries into an arm other than the goal arm; [27], [35]). Spatial learning, and reference and working memory, were assessed by comparing improvement in these measures across the 30 trials.

#### Retention

Retention of the location of the platform was tested 3 days after the spatial learning trials [27], [36]. To test retention, rats underwent a single trial identical to the spatial learning trials with the platform “invisible” just below the surface of the water such that rats had to recall the location from memory. For the retention trials, if a rat had not located the platform within 2 minutes it would be removed, however, no rat failed to locate the platform within 2 minutes.

### Data analysis

Latency to enter an arm, latency to locate the platform, search time, number of arm entries, and number of reference and working memory errors were natural log transformed to achieve normality. Three-trial means were calculated for all measures to reduce noise [37]. Learning and memory data were tested using repeated measures analyses of variance (RMANOVA) with stress treatment and time (three-trial means) as fixed effects. To determine whether adolescent-stress affected change across the spatial ability test, we calculated difference scores by subtracting the last three-trial mean from the first three-trial mean. Difference scores were compared using two-tailed t-tests. Retention was tested using t-tests to compare adolescent-stressed and unstressed rat performance. Two rats in the adolescent-stress treatment developed ulcerated tumors; they were removed from testing and are not included in any analyses. Analyses were run using IBM^®^ SPSS^®^ Statistics Version 21; values are reported as means ± standard error.

## Results

### Spatial abilities

**Fig 3:**
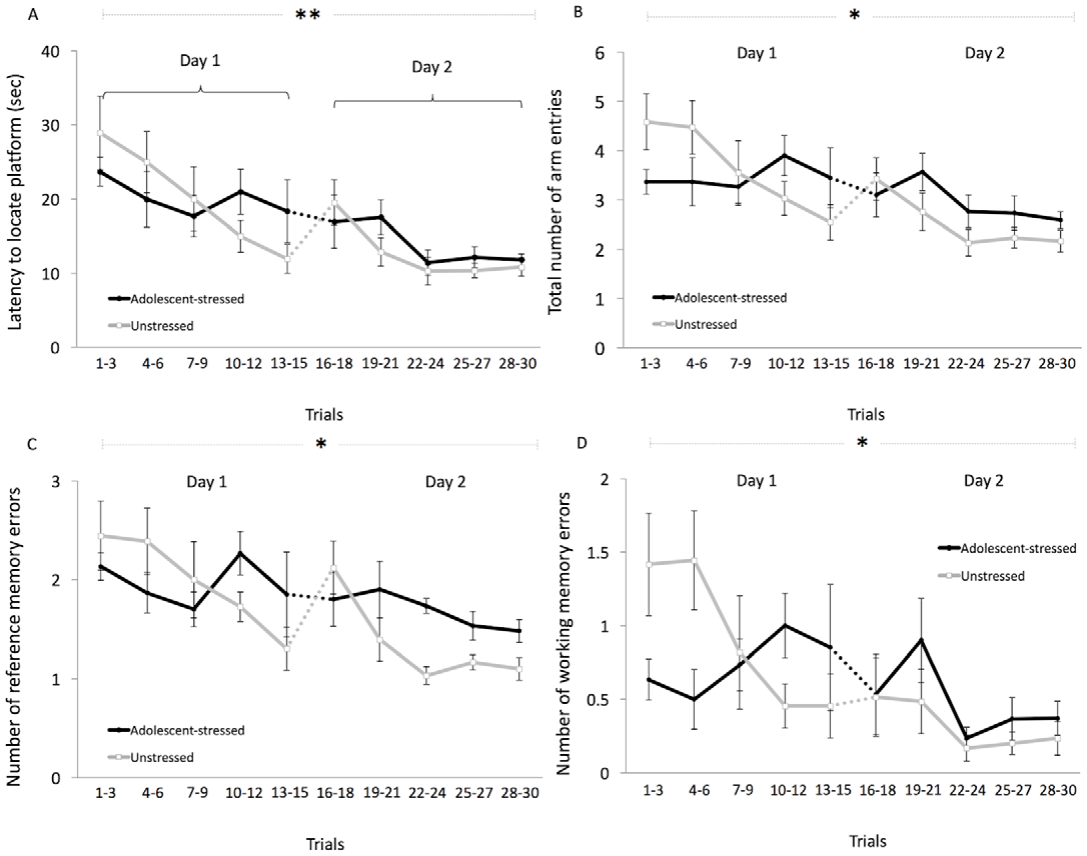
Spatial abilities test in adolescent-stressed and unstressed Sprague-Dawley male rats. The effects of adolescent-stress on latency to locate the platform (A), arm entries (B), reference memory (C), and working memory (D), means ± SE. *Indicates a significant time effect across all trials. **Indicates significant time and stress x time effects across all trials.

**Fig 4:**
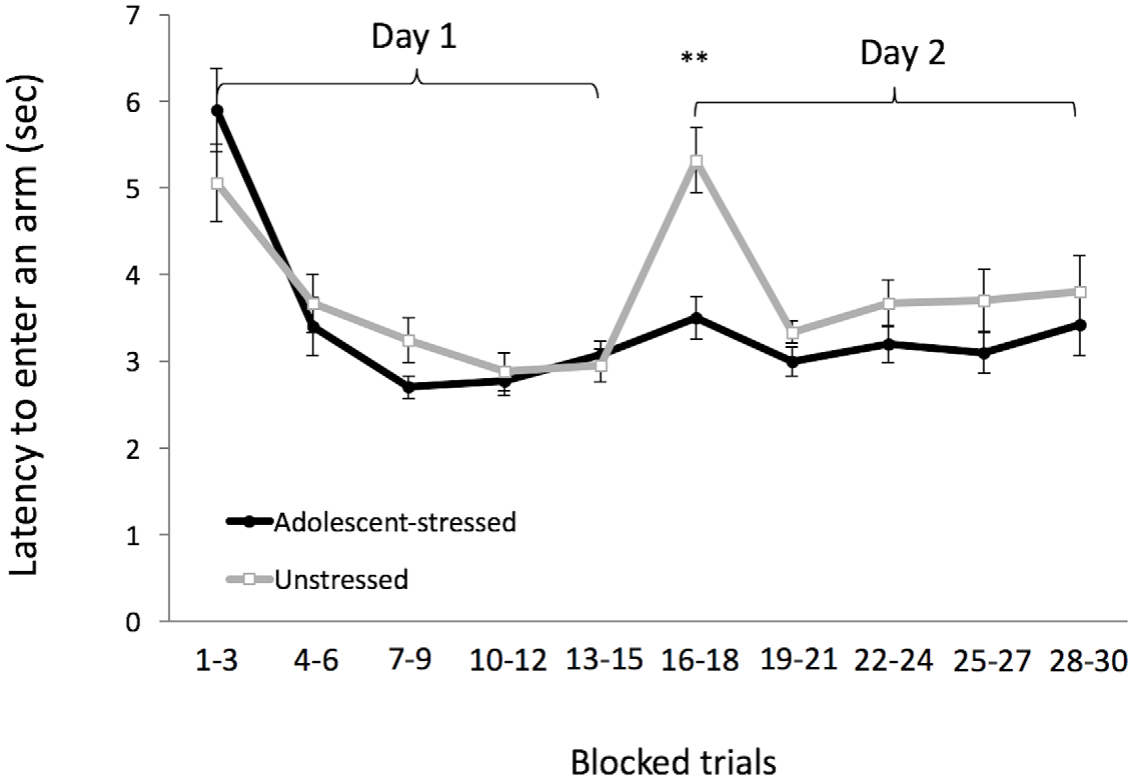
Latency to enter an arm across both days of radial arm maze training. Each point represents three averaged trials, means ± SE. **Indicates significant time and stress x time effects across all trials.

Exposure to stress during adolescence had no main effect on spatial abilities, including learning, working memory, and reference memory (Table 1, Fig 3). However, we found that unstressed rats reduced their number of arm entries more over time compared with adolescent-stressed rats, suggesting that adolescent-stressed rats were not improving their performance by incorporating spatial information with additional exposure to the task (Fig 3). Similarly, when we compared behaviors during the first block of spatial learning trials with behaviors during the last trial block, we found that adolescent-stressed animals showed a smaller change, compared with unstressed rats, in the number arm entries (23% vs. unstressed: 53%) and reference memory errors (31% vs. unstressed: 55%). This indicates that compared with unstressed rats, adolescent-stressed rats showed less improvement in their spatial learning performance over time, and did not refine their performance when given the opportunity to acquire additional information about their environment while unstressed rats did (Table 2). Thus, despite our findings that adolescent environment does not affect outcomes in spatial learning (or working and reference memory), it would appear that the rate of change in spatial learning performance may be modulated by developmental experiences such that adolescent-stress causes less flexibility in performance. Adolescent-stressed rats also exhibited a decreased latency to begin searching for a platform in the water maze task when returned to the maze after an overnight delay on the second day of trials compared with unstressed rats, suggesting that exposure to chronic stress in adolescence allowed the animals to react to subsequent reoccurring challenges more quickly (Fig 4).

**Table 1:** Spatial abilities in adolescent-stressed and unstressed male Sprague-Dawley rats.

*Measure*	*Effect of stress*	*Effect of time (trial block)*	*Stress x time interaction*
Latency to enter an arm	*F*_1,20_ = 2.598, *P* = 0.125	*F*_1,20_ = 12.223, *P* < 0.000*	*F*_1,20_ = 2.890, *P* = 0.003*
Latency to find the platform	*F*_1,20_ = 0.258, *P* = 0.618	*F*_1,20_ = 6.234, *P* < 0.000*	*F*_1,20_ = 0.777, *P* = 0.638
Search time	*F*_1,20_ = 0.331, *P* = 0.573	*F*_1,20_ = 38.460, *P* < 0.000*	*F*_1,20_ = 2.035, *P* = 0.173
Number of arm entries	*F*_1,20_ = 0.110, *P* = 0.744	*F*_1,20_ = 12.760, *P* < 0.000*	*F*_1,20_ = 1.986, *P* = 0.044*
Number of working memory errors	*F*_1,20_ = 0.401, *P* = 0.535	*F*_1,20_ = 8.422, *P* < 0.000*	*F*_1,20_ = 0.647, *P* = 0.755
Number of reference memory errors	*F*_1,20_ = 0.809, *P* = 0.381	*F*_1,20_ = 9.382, *P* < 0.00*	*F*_1,20_ = 1.299, *P* = 0.241

*Indicates significant at p < 0.05.

**Table 2:** Change across spatial ability test in adolescent-stressed and unstressed rats.

*Difference score (DS)*	*Effect of stress*
DS: Latency to enter an arm	*T*_20_ = 0.245, *P* = 0.811
DS: Latency to find the platform	*T*_20_ = 0.484, *P* = 0.635
DS: Search time	*T*_20_ = -0.514, *P* = 0.614
DS: Number of arm entries	*T*_20_ = -2.806, *P* = 0.015*
DS: Number of working memory errors	*T*_20_ = -1.891, *P* = 0.088
DS: Number of reference memory errors	*T*_20_ = -3.121, *P* = 0.008*

*Indicates significant at p < 0.05.

### Retention

**Fig 5:**
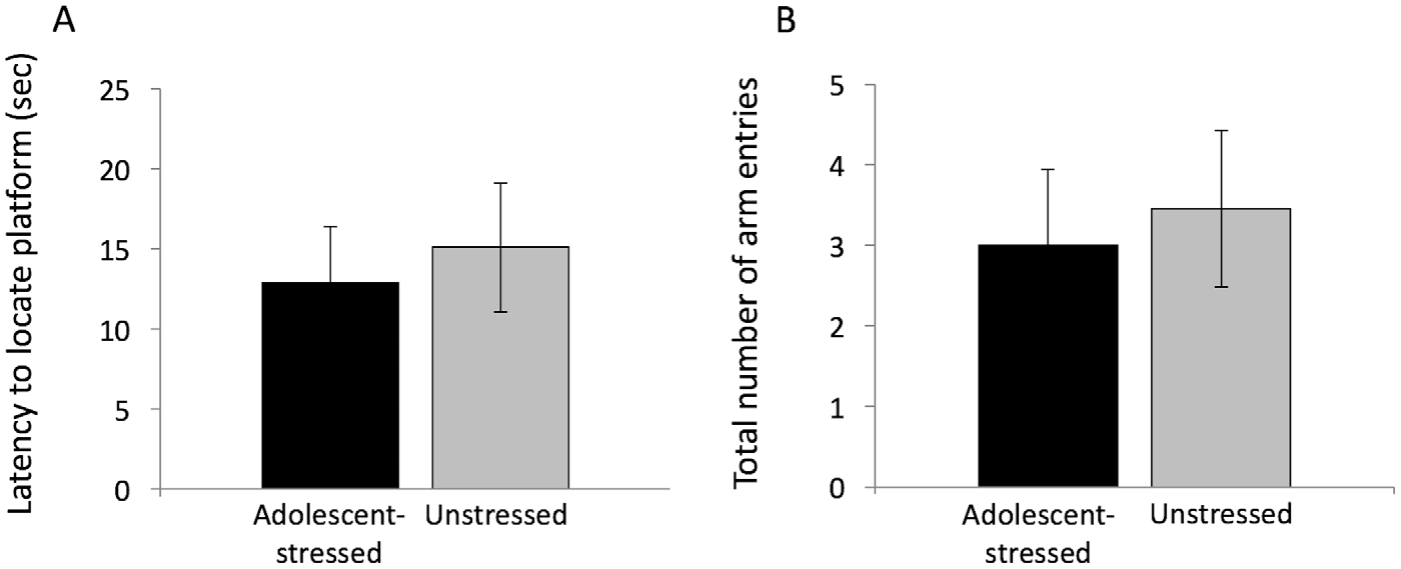
Retention of a spatial association in adolescent-stressed and unstressed Sprague-Dawley male rats. Retention of a platform location in a water maze 3 days after spatial association training was assessed using latency to locate the platform (A) and total number of arm entries before finding the platform (B), means ± SE.

We found no differences in retention of spatial information in adolescent-stress exposed and unstressed rats (Fig 5, Table 3).

**Table 3:** Retention in adolescent-stressed and unstressed rats.

*Retention*	*Effect of stress*
Latency to enter an arm	*T*_20_ = -0.433, *P* = 0.670
Latency to find the platform	*T*_20_ = -0.371, *P* = 0.715
Search time	*T*_20_ = -0.147, *P* = 0.886
Number of arm entries	*T*_20_ = -0.225, *P* = 0.824

## Discussion

Spatial abilities are dependent upon neural structures that mature during adolescence [30], [38]. Exposure to chronic stress in adolescence is thought to disrupt the maturation of these structures, possibly compromising their function later in life [21], [30]. In the current study, we tested the hypothesis that chronic stress in adolescence would impair adult spatial abilities and we found little support for our hypothesis. We show that adolescent-stress exposure had little effect on spatial learning, and found no evidence of impairments in spatial working memory, reference memory, or retention in adulthood. Conversely, we found that unstressed rats show greater improvement in spatial learning performance; over time they decrease the number of arms entered before finding the escape platform compared with adolescent-stressed rats. This suggests that adolescent-stressed rats do not improve their performance by incorporating spatial information with additional exposure to the task. Similarly, compared with unstressed rats, adolescent-stressed rats showed a smaller change in spatial learning measures; both in arms entered (23% vs. unstressed: 53%) and reference memory errors (31% vs. unstressed: 55%), suggesting that unstressed rats improved their performance over time, likely by acquiring spatial information, while adolescent-stressed rats chose a strategy that was not affected by additional information about their environment (Table 2). Interestingly, these differences in the degree of change in spatial learning measures did not affect overall performance between the treatments; possibly reflecting a change in strategy by adolescent stressed rats that results in fairly consistent performance over time compared to unstressed rats. Adolescent-stressed rats also exhibited a shorter latency to begin searching for a platform in the water maze task following an overnight delay compared with unstressed rats, suggesting that exposure to chronic stress in adolescence allowed animals to react to subsequent reoccurring challenges more quickly (Fig 4). Our results were unexpected because exposure to social instability, a chronic stress treatment, during adolescence has previously been shown to impair spatial learning [24] and spatial memory [23]. The contrast between earlier observations and those reported here suggests that the effects of adolescent-stress may be context-specific, and highlights the difficulty of generalizing results across systems and environments [16], [39].

Differences between the RAWM and the open arena tests used in prior studies of adolescent-stress could facilitate the use of different strategies and contribute to differences between the current results and those reported in earlier studies (Morris water maze: [24], [40]; object memory tests: [23], [33]). One explanation for our findings may lie in the use of alternate strategies in the RAWM by adolescent-stressed rats. The RAWM provides more visual and local cues than open arena tests, which may allow rats to employ an ‘associative learning strategy’ and decrease the need for hippocampally dependent cognitive maps of the environment, thereby masking possible spatial deficits [41], [42], [43]. Further, search strategies may differ between open arena tests and the more structured RAWM; in a RAWM, but not an open arena, it is possible to use a ‘working memory strategy’ by entering new arms until locating the platform, without re-entering previous arms [44]. Such an approach would not require retention across trials, just a ‘list-like’ working memory of which arms had been visited within a trial [44]. The current finding that unstressed rats show a greater decrease in errors related to reference memory compared with adolescent-stressed rats, but that stress condition does not affect changes in working memory (Table 2), allows for the possibility that adolescent-stressed rats used working memory to compensate for delays in reference memory, rather than a spatial learning strategy where a mental representation of the spatial environment is constructed and refined over time [45]. A ‘working memory strategy’ would also not be affected by delays between trials, and could explain why the adolescent-stressed animals in the current study appeared to show a smaller change in performance between the last set of trials on the first day and the first set of trials on the second day (separated by an overnight delay), compared with unstressed animals.

A shift in strategy [46], the flexibility of a strategy, or the ability to abandon an inefficient strategy [47] can result from exposure to stress. In addition to our current results, we have previously shown that the adolescent-stress procedures used here can induce strategy shifts. In Chaby et al. [29], we demonstrated that adolescent-stressed rats exhibit the same foraging performance as unstressed rats under standard testing conditions, but adolescent-stress changes foraging behaviors (including the number of patches visited and the latency to visit a patch). This suggests that adolescent-stress induces a change in foraging strategy without altering performance outcome. Additionally, although adolescent-stress does not affect the rate of appetitive associative learning, adolescent-stressed rats show increased decision making speed during associative learning training, again indicating a potential strategy change [12]. We suggest that these stress-induced strategy shifts could be adaptive in unpredictable or high threat contexts. If exposure to stress in adolescence signals that an animal should prepare for an environment where spatial cues will likely be unstable, or dangerous conditions where threat limits the amount of time an animal can spend acquiring spatial information, then it may be advantageous for adolescent-stressed animals to use spatial navigation strategies that enable rapid choices and do not rely on learning and environmental consistency [16]. Adjusting spatial strategies in response to environmental conditions has previously been shown in a teleost fish; stickleback that occupy unstable river environments are less likely to use visual landmarks compared with fish from more stable pond environments [9].

We also found that adolescent-stressed rats responded more quickly than unstressed rats when re-exposed to the potentially stressful cold water in the RAWM by beginning to search for the escape platform faster after an overnight delay between trials, which may be indicative of preconditioning effects [48], [49]. This finding also suggests that compared with unstressed rats, rats that experienced reoccurring challenges during the chronic adolescent-stress treatment may have a greater expectation of re-exposure to aversive stimuli that allows them to respond more quickly to reoccurring aversive conditions. An ability to engage in an aversive task more quickly could be beneficial in a context where faster action or escape would be advantageous. Given that chronic exposure to corticosteroids in adolescence can cause changes in impulsivity in adulthood, including increased impulsive choice and decreased impulsive action [50], it is also possible that changes in impulsivity may mediate changes in the latency to engage in the water maze task.

Overall, though we found little support of the hypothesis that chronic stress in adolescence impairs spatial ability in adulthood, the results indicate that adolescent-stress affects cognition in adulthood in a highly context-specific way. We suggest that exposure to adolescent-stress may cause a shift in strategy that affects behavior but results in equal performance, and increases reliance on working memory while decreasing the use of cognitive spatial maps. In an adverse environment where spatial cues are unstable or the presence of threat restricts the amount of time an animal can spend acquiring spatial information, it may be advantageous to reduce reliance on strategies that depend upon environmental consistency, like cognitive spatial maps, and instead favor strategies that allow rapid decision making. Our results emphasize the idea that stress in adolescence may have lasting effects on not only performance outcomes, but also on the strategies used to achieve potentially complex goals like navigating a spatial environment. Studies that further isolate different components of learning, such as place and spatial associative learning, would further refine these observations. Finally, our results suggest that experiencing reoccurring challenges during adolescence may allow animals to respond more quickly to subsequent reoccurring challenges, which could be valuable in a context where faster action would be advantageous.
